# Development and Evaluation of a Web-Based Platform for Dementia Caregivers: A Two-Phase Mixed-Methods Study

**DOI:** 10.1093/geroni/igaf122.4267

**Published:** 2025-12-31

**Authors:** Qiping Fan, Minh-Nguyet Hoang, Logan DuBose, Laura Kim, Louis Fisher, Shinduk Lee, Marcia Ory, Tokunbo Falohun

**Affiliations:** Clemson University, Clemson, South Carolina, United States; Texas A&M University, College Station, Texas, United States; Texas A&M University, College Station, Texas, United States; Texas A&M University, Texas A&M University, Texas, United States; Clemson University, Clemson, South Carolina, United States; University of Utah, Clemson, Utah, United States; Texas A&M University, College Station, Texas, United States; Texas A&M University, College Station, Texas, United States

## Abstract

Dementia caregivers face significant challenges, including managing financial, legal, and daily care responsibilities, yet existing resources often lack the personalization required to meet their complex needs. This study developed and evaluated Olera.care, a web-based platform designed to provide tailored educational and professional resources for dementia caregivers, using a two-phase mixed-methods design. Phase I included 30 family caregivers from Texas and employed a mixed-methods approach to identify caregiving challenges through qualitative interviews, develop and refine platform features, and pilot-test usability using the Mobile Application Rating Scale (MARS). Caregivers, predominantly aged ≥50 years (83%), women (77%), and White (83%), rated the platform highly for overall satisfaction (mean 4.57/5, SD 0.44), functionality (mean 4.46, SD 0.44), aesthetics (mean 4.58, SD 0.53), and personalized information quality (mean 4.76, SD 0.44). Phase II evaluated the platform usability and functionality among 65 caregivers from across the U.S. using the Technology Acceptance Survey (TAS), achieving an average score of 5.83/7 (SD 0.85), demonstrating strong usability and acceptance. Caregiver participants represented diverse demographics, with 69% White and 31% identifying as Black, Asian, Hispanic, or multiracial/ethnic. Care recipients exhibited varying levels of functional impairment (FAST levels 2–7d). Across both phases, caregivers emphasized the value of the platform in addressing caregiving challenges, particularly functional, legal, and financial needs. With its user-friendly design and personalized resources, Olera.care is a scalable, effective tool that supports dementia caregivers and addresses their growing needs. The study received funding support from the Small Business Innovative Research Program of National Institute on Aging.

